# Phytochemical fingerprinting of phytotoxins as a cutting-edge approach for unveiling nature’s secrets in forensic science

**DOI:** 10.1007/s13659-024-00484-6

**Published:** 2025-01-03

**Authors:** Nabil Zakaria, Ashraf S. A. El-Sayed, Mostafa G. Ali

**Affiliations:** 1 Phytochemistry lab, Botany and Microbiology Department, Faculty of Science, Zagazig University, Zagazig, 44519 Egypt; 2 Enzymology and Fungal Biotechnology Lab, Botany and Microbiology Department, Faculty of Science, Zagazig University, 44519 Zagazig, Egypt; 3 Botany and Microbiology Department, Faculty of Science, Benha University, Benha, 13518 Egypt; 4 Department of Food Science and Technology, The Ohio State University, Columbus, OH USA

**Keywords:** Forensic phytochemistry, Phytochemical fingerprinting, Plant toxins, Advanced chromatography

## Abstract

The integration of phytochemistry into forensic science has emerged as a groundbreaking frontier, providing unprecedented insights into nature's secrets through the precise application of phytochemical fingerprinting of phytotoxins as a cutting-edge approach. This study explores the dynamic intersection of phytochemistry and forensic science, highlighting how the unique phytochemical profiles of toxic plants and their secondary metabolites, serve as distinctive markers for forensic investigations. By utilizing advanced techniques such as Ultra-High-Performance Liquid Chromatography (UHPLC) and High-Resolution Mass Spectrometry (HRMS), the detection and quantification of plant-derived are made more accurate in forensic contexts. Real-world case studies are presented to demonstrate the critical role of plant toxins in forensic outcomes and legal proceedings. The challenges, potential, and future prospects of integrating phytochemical fingerprinting of plant toxins into forensic science were discussed. This review aims to illuminate phytochemical fingerprinting of plant toxins as a promising tool to enhance the precision and depth of forensic analyses, offering new insights into the complex stories embedded in plant toxins.

## Introduction

In the complex field of forensic science, a new discipline has emerged that intertwines botany and criminal investigation—forensic phytochemistry [1]. This growing field leverages plant-based evidence to illuminate mysteries that often evade traditional investigative techniques [2]. As we delve into the intersection of nature and crime, the importance of forensic phytochemistry becomes increasingly clear [3]. The exploration of phytochemical fingerprints emerges as a promising, cutting-edge method for unraveling the mysteries hidden within the toxic plants that populate our environment. Unlike traditional forensic analyses that primarily focus on human-derived evidence, forensic phytochemistry expands the investigative scope by incorporating the distinct chemical fingerprints imprinted on plant matter [4]. The field of forensic botany has been recognized since the mid-1900s, specifically when Arthur Koehler presented wood evidence in a court setting during the trial of the Charles Lindbergh baby kidnapping case [5].

The utilization of plants at both microscopic and macroscopic levels such as their organs (including leaves, stems, seeds, fruits, and roots), tissues (such as pollen grains, spores, fibers, and cork), and even their chemical components (including secondary metabolites, isotopes, DNA, and starch grains) as forensic evidence has been practiced since ancient times [6]. The Plantae kingdom encompasses approximately 400,000 species, with an additional 2,000 species documented annually, spans terrestrial and aquatic ecosystems and consist of multicellular, eukaryotic, and autotrophic organisms [7]. From leaves and pollen to seeds and extracts, plants carry a silent witness to their surroundings and interactions, becoming invaluable tools in the pursuit of justice [8–10]. Plant toxins are natural secondary metabolites produced by plants as a defense mechanism against herbivores, pathogens, and competing plants [11, 12]. While these toxins play an essential role in protecting plants, they can pose significant risks to humans when consumed or upon contact. Plant toxins vary widely in their chemical nature and effects on human health. Organic compounds, including alkaloids, terpenes, flavonoids, tannins, cardiac and cyanogenic glycosides, proanthocyanidins, phenylpropanoids, lignans, nitrogen-containing compounds, resins, oxalates, and certain proteins or amino acids, represent the main poisonous substances found in plants [12]. The release of these toxic biological agents is even considered as a bioterrorism [11]. Additionally, some poisonous plants are capable of accumulating inorganic compounds from the soil.

This study addresses the critical need for more refined and advanced methodologies in plant toxin analysis by exploring phytochemical fingerprinting. By gaining a comprehensive understanding of the unique chemical profiles of toxic plants, forensic scientists can better attribute toxins to specific botanical sources. From analyzing botanical fingerprints to examining real-world case studies, we navigate the intricate intersection of nature's secrets and forensic investigation, reshaping the landscape of criminal justice [13]. Unlocking the secrets encoded in phytochemical fingerprints not only enhances our understanding of toxic plant species but also contribute to more accurate and reliable forensic investigations. The potential implications are broad, with applications in criminal cases, environmental monitoring, and emergency response scenarios. This review utilizes state-of-the-art chromatographic and spectroscopic techniques, including Ultra-High-Performance Liquid Chromatography (UHPLC) and High-Resolution Mass Spectrometry (HRMS) to develop detailed phytochemical fingerprints. By integrating these advanced analytical methods, we aim to provide forensic scientists with a comprehensive and efficient tool for forensic scientists in the identification and analysis of plant toxins.

## Literature screening and data extraction

### Inclusion criteria

- Scientific Credibility: Priority was given to papers published in peer-reviewed journals recognized in the fields of forensic science and botany.
- Subject Relevance: Studies directly addressing phytotoxins and their applications in forensic science were selected.
- Publication Date: Recent publications were prioritized to ensure up-to-date information; however, foundational classic references were also included.

### Exclusion criteria

- Low-Quality Studies: Papers lacking reliable data or presenting unsupported experimental results were excluded.
- Irrelevance: Studies not directly related to the topic, such as those focusing on areas far removed from plant toxins or forensic science, were omitted.
- Overly Analytical Focus: References addressing purely analytical topics without practical applications in forensic science were not included.
- Non-Refereed Sources: Conference papers that were not peer-reviewed were excluded.

The selection procedure for the references used in this research is summarized as follows:

### Assimilation

- Keywords: forensic phytochemistry, phytochemical fingerprinting, plant toxins, advanced chromatography.
- Sources: PubMed, Scopus, Google Scholar, Web of Science.
- Publisher Websites: Elsevier, Wiley, Springer, Royal Society of Chemistry (RSC).
- Article Types: Research papers, case reports, books, and reviews (Total: 234).

### Screening:

Exclusions (n = 37): Duplicated publications (n = 12), studies not meeting selection criteria (n = 18), and articles unavailable for access (n = 7).

### Suitability:

Final database (n = 197): Research papers (n = 86), case reports (n = 6), books (n = 36), reviews (n = 69).

## Historical cases of plant poisoning

Forensic phytochemistry had made a significant impact beyond theoretical applications, demonstrated in real-world case studies that underscore its utility in criminal investigations [14, 15]. These cases vividly demonstrate how plant-based evidence can be a pivotal in solving crimes, offering a unique intersection between nature and forensic science. Throughout history, numerous cases of plant poisoning incidents have showcased the forensic importance of plant-related toxicology. Below are several notable historical incidents:

### Case study 1: the poisonous garden

In a high-profile poisoning case, forensic phytochemistry played a central role when investigators examined the victim's surroundings [14]. Analysis of plant samples from the crime scene revealed presence of toxic compounds unique to a rare plant species. This finding not only identified the poisonous agent but also traced it to a specific geographical origin [16], ultimately helping to apprehend the perpetrator. Plant-derived toxins have been used by criminals in cases involving homicide, theft, and rape cases. Toxicity can manifest in different forms, including skin irritation from direct contact, or internal poisoning through ingestion, absorption through the dermal layer or inhalation.

### Case study 2: Socrates’ death

The ancient Greek philosopher Socrates was sentenced to death in 399 BC by ingesting a cup of hemlock [17]. Hemlock (*Conium maculatum*) contains the potent neurotoxin coniine, which led to respiratory paralysis followed by Socrates' eventual death. This case is a well-known example of the intentional use of a plant toxin for execution.

### Case study 3: the Borgia family poisonings

The notorious Borgia family, active during the Italian Renaissance, was rumored to have used various toxic plants for political assassinations [18]. Nightshade (*Atropa belladonna*) which contain the alkaloid atropine, was one of the substances suspected to be involved. These poisonings highlight the historical use of plant toxins in political intrigue.

### Case study 4: locoweed poisoning in livestock

In North America, particularly in the western regions, locoweeds (*Astragalus* and *Oxytropis* genera) are known to cause poisoning in livestock [19]. Consumption of locoweeds by cattle and horses leads to a condition known as locoism, characterized by neurological symptoms. Forensic investigation into livestock deaths often focus on identifying the specific locoweed species responsible.

### Case study 5: water Hemlock poisonings

Water hemlock (*Cicuta* species) is considered one of the most toxic plants in North America [20]. Several accidental ingestions have resulted in poisoning. Notable victims include explorers Meriwether Lewis and Christopher McCandless. Forensic examination in such cases involve identifying the plant and its toxic components.

### Case study 6: Foxglove poisonings

Foxglove (*Digitalis purpurea*) contains cardiac glycosides, which can be fatal if ingested in large quantities [21]. Historical cases include both accidental ingestions and intentional poisonings. The digitalis compounds have medicinal uses, but improper administration can be dangerous. Forensic analysis helps detect the presence of foxglove toxins in the body.

### Case study 7: Cassava cyanide poisoning

Cassava (*Manihot esculenta*) contains cyanogenic glycosides, which can release hydrogen cyanide when metabolized [22]. In regions where cassava is a dietary staple, cases of cyanide poisoning have occurred due to improper processing or consumption of inadequately prepared cassava products. Forensic investigation in these instances involve detecting cyanide in post-mortem samples.

These historical cases underscore the critical role of plant-related toxicology in forensic investigations. Identifying specific plant toxins, understanding their mechanisms of action, and distinguishing between accidental and intentional poisonings are essential aspects of forensic analysis. Advances in analytical techniques over time have improved the detection and quantification of plant toxins, enhancing to the field of forensic toxicology.

## Toxic plants in forensic pathology

Toxic plants can play a significant role in forensic pathology, often influencing the determination of the cause of death [14]. These plants pose diverse challenges in forensic pathology, ranging from accidental poisonings to intentional acts of self-harm or homicide. Forensic pathologists, in collaboration with toxicologists and phytochemists, play a crucial role in identifying and understanding the implications of plant toxins during postmortem examinations. Comprehensive investigations and detailed documentation are essential to uncover the role of toxic plants in the cause of deaths and contribute to inform legal and public health outcomes. Below are several implications and considerations when dealing with toxic plants in forensic pathology:

### Accidental poisoning

*Ingestion of Toxic Plants* Accidental ingestion of toxic plants, especially by children or individuals unfamiliar with their toxicity, can lead to poisoning [23]. Forensic pathologists must evaluate the possibility of accidental ingestion when determining the cause of death.

### Suicidal poisoning

*Plant Toxins as Suicide Means* Some individuals may intentionally ingest toxic plants as a method of suicide [24]. The discovery of plant toxins in postmortem examinations raises questions about the intent behind the poisoning, requiring a detailed investigation.

### Homicidal poisoning

*Use of Plant Toxins in Homicides *In certain cases, plant toxins may be used as a means of homicide [25]. Forensic pathologists need to identify patterns that suggest intentional poisoning, including the presence of specific plant toxins.

### Identification of plant toxins

*Challenges in Identification* Identifying plant toxins in postmortem samples can be challenging due to factors such as postmortem changes, toxin degradation, and the need for specialized analytical techniques [26].

*Collaboration with Toxicologists* Forensic pathologists often collaborate with forensic toxicologists who employ advanced analytical methods to identify and quantify plant toxins in biological samples.

### Postmortem changes and decomposition

*Effect on Toxin Detection* Postmortem changes and decomposition processes can affect the detection of plant toxins [27]. Early postmortem examinations are preferred to minimize these challenges and obtain more accurate results.

### Clinical presentation vs. Autopsy findings

*Discrepancies in Symptoms* In cases of plant poisoning, there may be discrepancies between the clinical symptoms reported before death and the autopsy findings [28]. This emphasizes the importance of comprehensive toxicological analyses to confirm the presence of plant toxins.

### Histopathological examination

*Organ Damage* Plant toxins may cause specific histopathological changes in organs [29]. Histopathological examination of tissues can provide additional evidence about the effects of plant toxins on the body and assist in determining the cause of death.

### Documentation and case history:

*Detailed Case Documentation* Forensic pathologists must meticulously document the circumstances surrounding the death, including any information related to the ingestion or exposure to toxic plants [30]. This documentation is crucial for conducting comprehensive forensic analyses.

### Collaboration with phytochemists and public health concerns:

Collaboration with phytochemists becomes essential when dealing with plant-related poisonings. Botanists can help identify specific plant species and provide insights into the toxicity levels. The discovery of toxic plants in forensic pathology raises public health concerns, as identifying poisonous plants and assessing their potential risks can lead to preventive measures against accidental poisonings [31].

### Legal implications

*Forensic Testimony* Forensic pathologists may be required to provide expert testimony in legal proceedings [32]. Clear communication of findings, including the presence of plant toxins and their role in the cause of death, is crucial for legal clarity.

## Toxic principles of plant origin

The study of plant toxins in forensic science involves the identification, analysis, and interpretation of toxic substances derived from plants, particularly in the context of criminal investigations or legal proceedings [33, 34]. Phytotoxins (plant derived toxic principles) play a significant role in forensic cases related to poisoning, environmental crimes, and even biological warfare. It's important to note that the toxicity of plant compounds varies widely, and many plants that produce toxins are harmless when consumed in moderation or properly processed. Additionally, some plants with toxic components have been historically used in traditional medicine for therapeutic purposes. Plant toxins are systematically categorized into four distinct classes based on their lethal oral dose as ascertained through rat experimentation: class Ia: extremely hazardous (LD_50_ of 5 mg or less per kg of body weight); class Ib: highly hazardous (LD_50_ of 5 to 50 mg/kg of body weight); class II: moderately hazardous (LD_50_ of 50 to 500 mg/kg of body weight); and class III: slightly hazardous (LD_50_ of 500 mg and above per kg of body weight) [35, 36]. Consequently, proper identification and understanding of these toxins are crucial for ensuring safety and avoiding adverse effects. Forensic plant toxins can be classified into different categories based on their chemical nature and the effects they have on the human body.

### Alkaloids

Alkaloids are naturally occurring organic compounds that contain basic nitrogen atoms with diverse structural forms. Some alkaloids have stimulant properties, while others are toxic to the nervous system, potentially leading to poisoning or even death at higher doses [37, 38]. Plant-derived toxic alkaloids of forensic significance belong to various classes, each characterized by unique structural features and toxicity profiles. Table. 1 provides an overview of the chemistry and occurrence of plant-derived toxic alkaloids that are employed as toxicological markers in forensic science.

**Table 1:** The chemistry and occurrence of plant-derived toxic alkaloids of forensic significance

Chemical nature	Toxicological marker	Natural source			Toxicity class*	References
		Hazardous plant parts	Toxic plant species	Plant family		
Pyridine alkaloids	Nicotine Cotinine	Leaves	Tobacco plant *Nicotiana tabacum*	Solanaceae	Ib	[37]
Piperidine alkaloids	Anabasine Anatabine Nornicotine	Leaves	Tobacco plant *Nicotiana glauca*	Solanaceae	Ib	[119]
	Coniine *N*-methyl-coniine Coniceine γ-coniceine Cicutoxin Conhydrine Pseudoconhydrine	Leaves Stems Seeds	Poison-hemlock *Conium maculatum*	Apiaceae	Ia	[120]
	Sedamine Sedinine	All parts	Common stonecrop *Sedum acre*	Crassulaceae	II	[121]
Pyrrolizidine alkaloids	Symphytine Echimidine Allantoin	All parts Roots	Comfrey *Symphytum officinale*	Boraginaceae	II	[122]
	Senecionine	All parts especially Flowers	Tansy ragwort *Senecio jacobaea*	Asteraceae	II	[123]
	Lycopsamine	All parts	Common bugloss *Anchusa officinalis*	Boraginaceae	II	[124]
	Colchicine	All parts, especially seeds, bulbs	Autumn crocus *Colchicum autumnale*	Liliaceae	Ia	[125]
	Temuline Loline	All parts	Poison darnel *Lolium temulentum*	Poaceae	Ib-II	[126]
Quinolizidine alkaloids	Nupharine Desoxynupharidine	All parts	yellow pond lily *Nuphar lutea*	Nymphaeaceae	II	[127]
	Lupanine Sparteine	Seeds	Lupin *Lupinus polyphyllus* Broom *Cytisus scoparius*	Fabaceae	Ib-II II	[128]
	N-methylcytisine Cytisine	All parts especially Seeds	Spanish broom *Spartium junceum*	Fabaceae	Ib	[129]
	Anagyrine	All parts	Dyer's greenweed *Genista tinctoria*	Fabaceae		[130]
Benzophenanthridine alkaloids	Chelidonine Chelerythrine	All parts, reddish latex	Celandine *Chelidonium majus*	Papaveraceae	Ib-II	[131]
	Sanguinarine	Seeds	Prickly-poppy *Argemone mexicana*	Papaveraceae	Ia	[132]
Benzylisoquinoline alkaloids	Protopine	Rhizomes Roots	Three-leaf corydalis *Corydalis ternata*	Papaveraceae	II	[132]
Isoquinoline alkaloids	Berberine	All parts, especially latex	Goldenseal *Hydrastis canadensis*	Ranunculaceae	II	[132]
			Oregon grape *Berberis aquifolium*	Berberidaceae	II	
	Rhoeadine Protopin		Common poppy *Papaver rhoeas*	Papaveraceae	II	[133]
	Galanthamine Lycorine Tazettine	Bulbs	Spring Snowflake *Leucojum vernum*	Amaryllidaceae	II	[134]
	Glaucine Magnoflorine Protopine Sanguinarine	All parts	yellow hornpoppy *Glaucium flavum*	Papaveraceae	II	[135]
	Bulbocapnine Protoberberine Apomorphine	All parts, especially tubers	Hollowroot *Corydalis cava*	Papaveraceae	II	[136, 137]
	Opium Morphine Codeine Thebaine Noscapine	Latex of seeds	The poppy plant *Papaver somniferum*	Papaveraceae	Ib	[38]
Steroidal alkaloids	Buxine Cyclobuxine	Leaves	Common box *Buxus sempervirens*	Buxaceae	Ib	[138]
	Protoveratrine A Protoveratrine B Germerine Cyclopamine	Roots	False hellebore *Veratrum album*	Melanthiaceae	Ia	[139]
	Imperialine Tuliposide A Tulipalin A	Bulbs	Crown imperial *Fritillaria imperialis*	Liliaceae	II	[140]
Tropane alkaloids	Atropine	Leaves Flowers Roots Seeds	Deadly nightshade *Atropa belladonna*	Solanaceae	Ia	[119, 141]
	Hyoscyamine		Black henbane *Hyoscyamus niger*			
	Scopolamine		jimsonweed *Datura stramonium*			
	Cocaine	Leaves	Coca Plant *Erythroxylum coca*	Erythroxylaceae	Ia	[142]
Terpene alkaloids	Aconitine Mesaconitine Hypaconitine Jesaconitine Napelline	Roots	Monkshood *Aconitum* species	Ranunculaceae	Ia	[143, 144]
	Lycoctonine Delcosine	Seeds	Forking larkspur *Consolida regalis*	Ranunculaceae	Ia	[145]
	Delphinine Nudicauline Staphisine Ajacine	Seeds	Candle larkspur *Delphinium elatum*	Ranunculaceae	Ib	[146]
Indole alkaloids	Strychnine Brucine Vomicine Protostrychnine Strychnine-N-oxide	Seeds	*Strychnos nux-vomica*	Loganiaceae	Ia	[147]
	Ergotamine Ergotoxine	Seeds	*Ipomoea coerulea*	Convolvulaceae	Ib	[148]
	Vincamine	Aerial parts	Lesser periwinkle *Vinca minor*	Apocynaceae	III	[149]
Indolizidine alkaloids	Swainsonine	Seeds Leaves Flowers	Locoweed *Astragalus molissimus*	Fabaceae	II	[150]
Steroidal glycoalkaloids	Soladulcidine, Solanine, Solasodine, Chaconine	Green fruits Leaves	Potato *Solanum tuberosum* Tomato *S. lycopersicum* Black nightshade *S. nigrum*	Solanaceae	Ib-II	[151]
Tryptamine alkaloids	Psilocybin Psilocin Baeocystine	Fruiting body	Magic mushroom *Psilocybe azurescens*	Strophariaceae	II	[152]
β-keto-amphetamine alkaloids	Cathine Cathinone Norephedrine	Leaves	Khat plant *Catha edulis*	Celastraceae	Ib	[153]

^*^ Class Ia: extremely hazardous; Ib: highly hazardous; II: moderately hazardous; III: slightly hazardous as adapted from Wink, 2009 [121]

### Glycosides

Glycosides are compounds that consist of a sugar molecule linked to another non-sugar moiety (aglycone) such as cyanogenic glycosides (found in cassava), which release hydrogen cyanide upon metabolism, posing a significant toxicity risk, cardiac glycosides (found in oleander), which affect heart function, and glucosinolates (found in cruciferous vegetables), which can disrupt thyroid function. Another class, saponins, are amphipathic glycosides found in beans, asparagus, and some medicinal plants. Large quantities of saponins can disrupt cell membranes and may have hemolytic effects [39]. Different classes of glycosides of forensic significance are listed in Table 2.

**Table 2:** The chemistry and occurrence of plant-derived toxic glycosides of forensic significance

Chemical nature	Toxicological marker	Natural Source			Toxicity class*	References
		Hazardous plant parts	Toxic plant species	Plant family		
Cyanogenic Glycosides	Aroin	Stems	*Arum cyrenaicum* *Arum italicum*	Araceae	Ia	[154]
	Amygdalin Prunasin	Seeds	Bitter almond *Prunus dulcis*	Rosaceae	Ib-II	[155]
	Hydrocyanic acid	Roots	Cassava *Manihot esculenta* *Manihot utilissima*	Euphorbiaceae	Ib	[156]
	Linamarin Lotaustralin					[157]
	Dhurrin	grains	Great Millet *Sorghum bicolor*	Poaceae	Ib	[158]
Cardiac Glycosides (Steroidal Glycosides)	Cerberin	Seeds	Suicide Tree *Cerbera odollam*	Apocynaceae	Ia	[33, 159]
	Thevetin A, B Thevetoxin Neriifolin Peruviside Ruvoside	Seeds	Yellow Oleander *Thevetia peruviana*	Apocynaceae	Ia	[160]
	Calotoxin, Calotropins DI Calotropins DII	Latex in Leaves Stem Bark Fruits	Crown flower *Calotropis gigantea*	Asclepiadaceae	Ia	[161]
	Oleandrin Oleandrigenin	Leaves Stems	Oleander *Nerium oleander*	Apocynaceae	Ia	[162]
	Cardenolides (Digoxin-Digitoxin- Lanatoside)	Leaves	Foxglove *Digitalis purpurea*	Plantaginaceae	Ia	[163]
	Convalatoxin	Leaves Flowers	Lily of the valley *Convallaria majalis*	Asparagaceae	Ib	[164]
	Adonitoxin	All parts	Yellow Pheasant's Eye *Adonis vernalis*	Ranunculaceae	Ib	[165]
	Proscillardin A	Bulbs	Sea squill *Scilla maritima*	Asparagaceae	Ib	[6]
	Hellebrim, Hellebrigenin	Rhizomes	Black hellebore *Helleborus niger*	Ranunculaceae	Ia	[166]
Alcoholic Glycosides	Salicin Salicilin	Leaves Flowers Roots	*Primula* spp.	Primulaceae	III	[167]
Antraquinone Glycosides	Aloin A, B Antron Anthranol	Latex in Leaves	*Aloe* sp*.*	Liliaceae	II	[168]
Hydroquinone Glycosides	Arbutin	All parts	Leather bergenia *Bergenia crassifolia*	Saxifragaceae	Ib	[169, 170]
Saponin Glycosides	Tigonin	All parts	*Digitalis lanata*	Plantaginaceae	Ia	[171]
	Gitonin	All parts	*Digitalis purpurea*		II	
	Triterpene saponins	Rhizomes Roots	*Primula* sp*.*	Primulaceae	II-III	[172]
	Saponariosides A, B	All parts	*Saponaria officinalis*	Caryophyllaceae	II	[173]
	Aescin (escin)	Seeds (Conkers)	Horse chestnut *Aesculus* spp.	Sapindaceae	II	[174]
	Taxine A,B,C Taxicin I, II	Leaves, Seeds Bark	Yew plant *Taxus* spp.	Taxaceae	Ia	[175]
Thioglycosides (Glucosinolates)	Gluco-tropeolin	Leaves	Nasturtium *Tropaeolum majus*	Tropaeolaceae	III	[176]
	Sinigroside	Seeds	Mustard *Brassica* sp.	Brassicaceae	Ia	[177]
Triterpine glycosides	Passiflorine	Leaves	Passion flower *Passiflora edulis* *Passiflora incarnata*	Passifloraceae	II-III	[178]

^*^ Class Ia: extremely hazardous; Ib: highly hazardous; II: moderately hazardous; III: slightly hazardous as adapted from Wink, 2009 [121]

### Proteins

Certain plant-derived proteins such as Toxalbumin and Mucunain, act as toxins by interfering with cellular functions or causing damage to organs [40, 41]. Lectins (e.g., ricin from castor beans and phytohemagglutinin from red kidney beans), can interfere with protein synthesis or cause agglutination of red blood cells, leading to toxicity [42]. Protease inhibitors (e.g., trypsin inhibitors from soybeans and chymotrypsin inhibitors from potatoes), can interfere with protein digestion, potentially causing nutrient deficiencies over time [43]. These proteins and other toxic compounds are detailed in Table 3. Additional classes of plant toxins including phenols, anthraquinones, Tannins, sesquiterpene, sesquiterpene lactones and cannabinoids are summarized in Table 4.

**Table 3:** The chemistry and occurrence of plant-derived toxic proteins and amino acids of forensic significance

Chemical nature	Toxicological marker	Natural Source			Toxicity class*	References
		Hazardous plant parts	Toxic plant species	Plant family		
Proteins	Toxalbumin	Seeds	Hondala *Adenia palmata*	Passifloraceae	Ia	[41]
	Mucunain	Seeds	Velvet bean *Mucuna prurita*	Fabaceae	Ib	[179]
Lectins	Arbin	Seeds	Rosary pea *Abrus precatorius*	Fabaceae	Ia	[40]
	Ricin	Seeds	Castor bean *Ricinus communis*	Euphorbiaceae	Ia	[42]
	Mistletoe	Leaves	Mistletoe *Viscum album*	Santalaceae	II	[180]
	Curcin	Seeds	*Jatropha curcas*	Euphorbiaceace	Ia	[181]
	Phytohemagglutinin	Seeds	Red kidney beans* Phaseolus vulgaris*	Fabaceae	II	[182]
	Crotine	All parts	*Croton* sp.	Euphorbiaceae	Ia	[183]
	Snow-drop lectine	Bulb	Snowdrop *Gallanthus nivalis*	Amaryllidaceae	II	[184]
Non-protein amino acid	Azetidine 2-carboxylic acid	Roots	*Convallaria majalis*	Asparagaceae	Ib	[185]

^*^Class Ia: extremely hazardous; Ib: highly hazardous; II: moderately hazardous as adapted from Wink, 2009 [121]

**Table 4:** The chemistry and occurrence of other plant-derived toxins of forensic significance

Chemical nature	Toxicological marker	Natural source			Toxicity class*	References
		Hazardous plant parts	Toxic plant species	Plant family		
Phenolic compounds	Hydroquinone	Seeds	*Xanthium strumarium*	Asteraceae	II	[186]
	Salligenin	Roots	*Primula* sp.	Primulaceae	II	[187]
Benzopyran	Coumarins	Peel	*Citrus* sp.	Rutaceae	II	[188]
	Chromones	Seeds	*Angelica arhangelica*	Apiaceae	II	[189, 190]
	Flavonoids Flavan, Flavone Isoflavone Catechins	All parts	*Euphorbia* sp.	Euphorbiaceae	II	[39, 128, 191]
Antraquinones	Hypericin	Flowers Leaves	*Hypericum perforatum*	Hypericaceae	II	[192]
Sesquiterpene	Picrotoxin	Seeds	Poison berry *Anamirta cocculus*	Menispermaceae	Ib	[193]
Sesquiterpene lactone	Parthenin	Pollen Trichomes	Parthenium *Parthenium hysterophorus*	Asteraceae	II	[55]
Carboxylic acid	Oxalic acid and its salt, Raphides	Leaves Roots	Dumb cane *Dieffenbachia seguine*	Araceae	III	[194]
Polyhydroxy alcohol	Tremetol	Leaves	White snakeroot *Ageratina altissima*	Asteraceae	Ib	[195]
Polyketides	Cicutoxin	Roots	Water hemlock *Cicuta virosa*	Apiaceae	Ib	[196]
Cannabinoids	Tetra-hydro Cannabinol	Trichomes	Marijuana *Cannabis sativa*	Cannabaceae	Ib	[197]

^*^ Class Ib: highly hazardous; II: moderately hazardous; III: slightly hazardous as adapted from Wink, 2009 [121]

### Phenols

Phenols are secondary metabolites produced by plants that serve as chemical defenses against herbivores, pathogens, and various environmental stressors. As plant toxins, phenols play a crucial role in deterring herbivores by affecting their digestion, growth, and overall health. These compounds can directly inhibit digestive enzymes, reducing nutrient absorption and making plant tissues less digestible [44]. This mechanism makes phenol-rich plants less palatable and nutritious for herbivores, which may deter them from repeated feeding.

Different types of phenols have distinct toxic effects. For example, tannins (a subset of phenols) can bind to dietary proteins, forming complexes that lower protein digestibility [45]. Some phenols can also interfere with cellular functions in herbivores, causing oxidative stress and cellular damage when ingested in large amounts [46, 47]. Moreover, phenols may cause bitter flavors or even adverse physiological responses, further protecting the plant. In essence, phenols function as natural toxins that allow plants to enhance their survival by reducing herbivory and protecting against disease, thereby contributing significantly to plant defense strategies.

### Tannins

Tannins are phenolic compounds widely found in plants and play a key defensive role, acting as plant toxins against herbivores. Hydrolysable tannins and condensed tannins (proanthocyanidins) represent two distinct classifications within the realm of these compounds. The two categories exhibit notable differences in their nutritional properties and toxicological implications. Condensed tannins exert a more significant impact on digestibility reduction in comparison to hydrolysable tannins, whereas the latter may induce diverse toxicological responses due to hydrolysis occurring within the rumen [48]. Tannins reduce digestibility and can damage the digestive systems of animals that consume plants rich in these compounds by binding to dietary proteins and forming complex compounds that hinder their digestion and absorption. This binding inhibits digestive enzymes, such as pepsin, making tannin-rich plants less nutritious for ruminants [45, 49].

In this way, tannins serve as a natural plant toxin and a defensive strategy, deterring herbivores from fully benefiting from plant nutrients, and in some cases, causing toxicity or discouraging them from consuming tannin-rich plants.

### Anthraquinones

Different anthraquinones such as Hypericin found primarily in *Hypericum perforatum* known for its phototoxic properties [50]. it can induce harmful effects when exposed to light, particularly ultraviolet (UV) light. When hypericin absorbs light, it can enter an excited state and generate reactive oxygen species (ROS). These highly reactive molecules can damage cellular components, including lipids, proteins, and DNA, leading to oxidative stress and cell death [51, 52].

### Sesquiterpene

Sesquiterpenes such as Picrotoxin is derived from the seeds of the plant *Anamirta cocculus*. It is more widely recognized for its neurotoxic properties [53]. Picrotoxin is known to act as a non-competitive antagonist of the gamma-aminobutyric acid (GABA) receptor. GABA is a major inhibitory neurotransmitter in the central nervous system. By inhibiting GABA receptor function, picrotoxin disrupts normal neuronal signaling, leading to increased neuronal excitability and potential seizures [53, 54].

### Sesquiterpene lactone

Such as Parthenin found primarily in *Parthenium hysterophorus.* It has gained attention not only for its phytotoxic and toxic effects on animals but also for its potential in vitro and in vivo genotoxicity [55]. Studies have shown that parthenin can induce DNA damage through the formation of reactive oxygen species (ROS), which can lead to oxidative stress and subsequent harm to DNA. Oxidative damage can result in single- and double-strand breaks, base modifications, and cross-linking of DNA strands [55]. Parthenin exposure has been associated with increased rates of chromosomal aberrations in cultured cells. These abnormalities can result from improper DNA repair mechanisms in response to damage, leading to further genomic instability [56, 57].

### Cannabinoids

Cannabinoids are a diverse class of chemical compounds found primarily in the cannabis plant (*Cannabis sativa*), known for their psychoactive and therapeutic properties [58]. Among the various cannabinoids, tetrahydrocannabinol (THC) that binds to cannabinoid receptors in the brain and body, leading to a range of effects, including euphoria, altered perception, and relaxation [59]. Tetrahydrocannabinol (THC) exemplifies the dual nature of cannabinoids, as both a beneficial therapeutic agent and a potential plant toxin. Understanding its mechanisms of toxicity and the contexts in which these effects arise is essential for safe use, particularly in medical and recreational settings [60].

## Phytochemical fingerprints: nature's unique identifier in forensic phytochemistry

Botanical fingerprints represent a groundbreaking concept in forensic phytochemistry, offering a distinctive and powerful means of identifying plant-based evidence. Analogous to human fingerprints, these botanical imprints capture unique chemical signatures of individual plant species, providing investigators with a powerful tool for tracing origins, movements, and connections involved in criminal investigations [2, 3].

### Chemical diversity in plants

Plants produce a vast array of secondary metabolites, resulting in a wide range of chemical compounds [61, 62]. These include alkaloids, flavonoids, and terpenoids, which contribute to the distinct chemical profiles that characterize each plant species [8]. Forensic scientists utilize this chemical diversity to create phytochemical fingerprints, akin to the genetic code that differentiate each plant apart.

### Geographical signatures

One of the remarkable aspects of phytochemical fingerprints is their ability to carry geographical information. Different regions yield distinct environmental conditions that influence the composition of plant secondary metabolites. By analyzing these chemical variations, investigators can deduce the geographic origin of plant samples. These insights can provide crucial information regarding crime scene locations or the movement of evidence [16, 63]. For example, chemical profiling of terpenoids and phytocannabinoids, can help to elucidate the geographic origin of confiscated marijuana [64]. In criminal investigations, these phytochemical fingerprints serve as a bridge linking crime scenes to suspects or connecting different crime scenes. A unique combination of plant compounds found at different locations can suggest a common origin or shared environmental factors, potentially tying together different aspects of an investigation.

### Temporal dynamics

Phytochemical fingerprints are not static; they evolve over time and in response to environmental changes [65, 66]. This temporal aspect allows forensic experts to trace the age of plant materials, heling establish event timelines, corroborate alibis, or uncover long-buried evidence in historical cases.

## Phytochemical fingerprinting techniques in plant toxin detection

Modern techniques used in phytochemical fingerprinting play a crucial role in the forensic detection and quantification of plant toxins. These methods provide accurate and sensitive measurements, helping forensic scientists to identify specific toxins, determine their concentrations, and establish connections between plant exposure and adverse health effects. Two of the most widely used techniques in plant toxin analysis are chromatography and mass spectrometry.

### Chromatography

Chromatography is a separation technique that exploits the different affinities of compounds between a mobile phase (liquid or gas) and a stationary phase (solid or liquid) [67]. The separation is based on factors such as molecular size, polarity, or charge [68].

- Gas Chromatography (GC): This technique is effective for analyzing volatile compounds [69], and plant toxins that can be vaporized without decomposition are often analyzed using GC.
- Liquid Chromatography (LC): Suitable for a wide range of compounds, including non-volatile and polar substances [70]. High-Performance Liquid Chromatography (HPLC) is a common subtype used in plant toxin analysis.

Chromatography is commonly employed for separating and isolating plant toxins from complex matrices, such as biological samples (e.g., blood, urine) or plant materials.

### Mass Spectrometry (MS)

Mass spectrometry is a technique that measures the mass-to-charge ratio of charged particles. It involves ionizing the sample and then analyzing the resulting ions based on their mass and charge [71, 72].

- Gas Chromatography-Mass Spectrometry (GC–MS): Integrates the separation capabilities of GC with the sensitivity and specificity of MS [73]. It is commonly used for volatile plant toxins.
- Liquid Chromatography-Mass Spectrometry (LC–MS): Combines the separation power of LC with the mass analysis of MS [74]. It is suitable for a broader range of compounds, including polar and non-volatile plant toxins.

Mass spectrometry is employed for the qualitative and quantitative analysis of plant toxins. It helps identify specific compounds by their mass spectra, providing valuable information about the molecular structure and aiding in the confirmation of toxin presence.

For instance, the aqueous extracts of ten plant samples; *Tussilago farfara*, *Senecio vulgaris*, *Lithospermum officinale*, *Anchusa officinalis*, *Echium italicum*, *Heliotropium europaeum*, *Symphytum officinale*, *Eupatorium cannabinum*, *Borago officinalis* and *Petasites hybridus*, collected from Orto botanicodellaScuola Medica Salernitana, located in Salerno, Italy, Salerno, Italy, were subjected to analysis by Ultra-high performance liquid chromatography-MS/MS (UPLC − MS/MS). This MS-based approach successfully identified 88 pyrrolizidine alkaloids (PAs) across a total of 282 samples, achieving an identification limit of 0.6 − 30 *μ*g kg^−1^ [75].

Kalb et al., [76] employed MS-based methods to detect and quantify two protein toxins: ricin and RCA120. The developed method was successfully detected the two protein toxins in nine samples of castor bean plants. However, samples with a concentration of 0.414 ng/mL could not be detected. Thirty plant toxins belonged to five predominant groups of plant toxins, which include terpenoids, alkaloids, flavonoids, steroids and aromatic polyketides, were successfully detected using reversed phase liquid chromatography coupled with high-resolution mass spectrometry (LC − HRMS) with 40 times higher detection sensitivity than that of preceding techniques [77].

### Nuclear magnetic resonance (NMR) spectroscopy

NMR spectroscopy measures the interactions between nuclear spins and an external magnetic field, providing information about the chemical structure and molecular dynamics of compounds [78]. In plant toxin analysis, NMR is used to confirm the chemical structure of identified compounds, complementing mass spectrometry by providing additional structural details [79]. NMR-based metabolic profiling techniques were employed to track changes in many plant metabolites for quality control and for detecting adulteration of products [80]. NMR spectroscopy was employed to describe the structures of large toxic plant proteins such as ricin from the castor bean plant [81]. The high-resolution 3D structure of viscotoxin A3, a phytotoxin found in from *Viscum album* L., has been determined in solution by ^1^H-NMR spectroscopy and deposited in the Protein Data Bank under the id. code 1ED0 [82].

### Enzyme-linked immunosorbent assay (ELISA)

ELISA is an immunological technique that employs antibodies to detect the presence of specific antigens, including plant toxins [83]. It is often used as a rapid and cost-effective screening method for detecting the presence of plant toxins in samples. While it may not offer the same specificity of chromatography and mass spectrometry, ELISA serves as a valuable preliminary tool for large-scale analyses.

These analytical techniques, when used in combination, offer a comprehensive approach for plant toxin detection and quantification in forensic investigations. Advances in instrumentation, sensitivity, and data analysis methods continue to enhance the accuracy and reliability of these techniques.

## Forensic investigation protocols

**Fig. 1:**
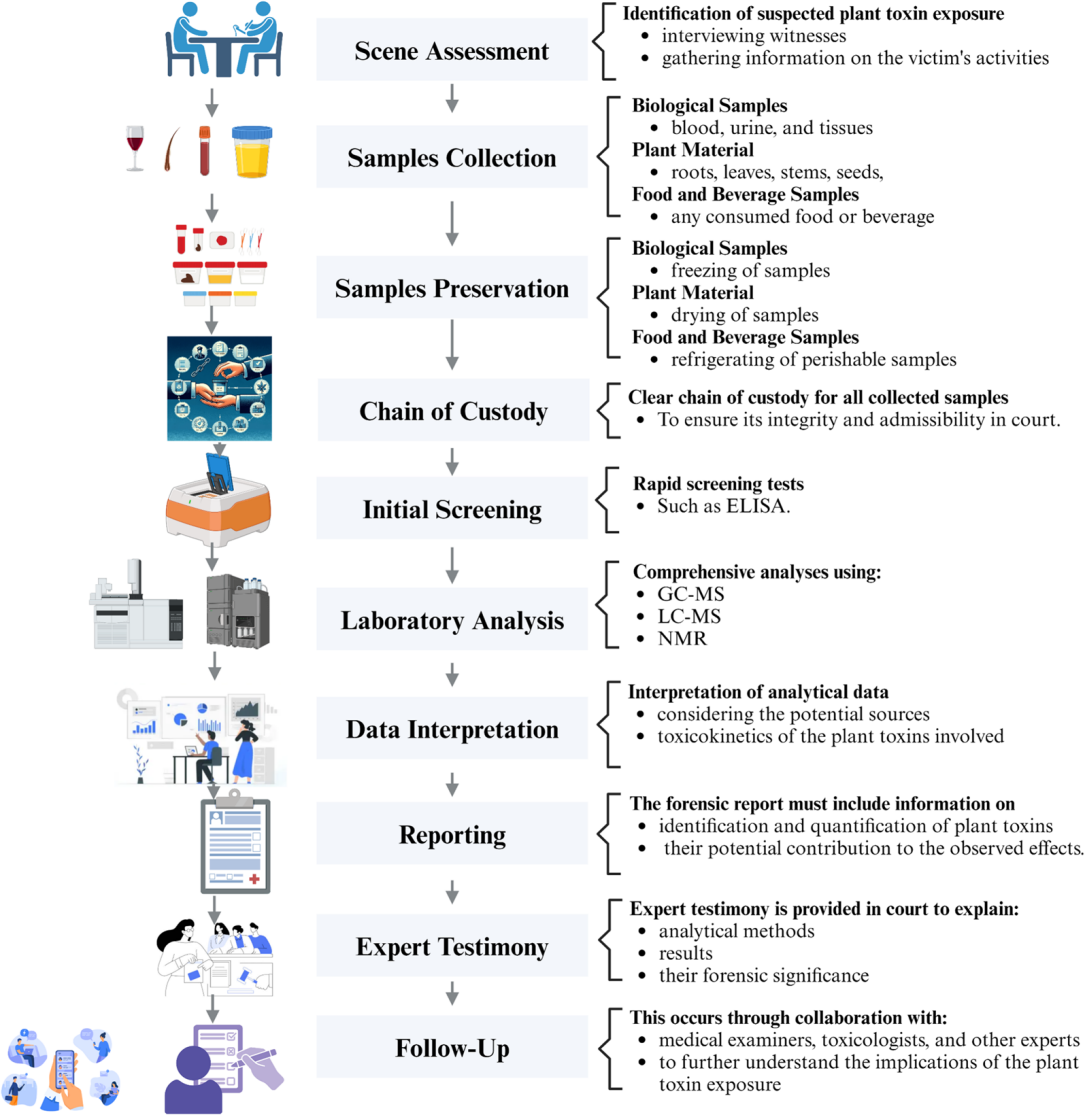
Forensic investigation protocols: From samples collection till conclusion about the reasons of suspected death

The forensic investigation of suspected plant toxin poisoning requires a systematic approach to ensure the proper collection, preservation, and analysis of relevant samples [84]. Below is a general protocol for conducting such forensic investigations and a flow chart highlighting these procedures is shown in Fig. 1:

### Scene assessment

Identification of suspected plant toxin exposure involves interviewing witnesses, gathering information on the victim's recent activities, diet, and exposures and finally searching for the presence of plants or plant-based products in the environment that could be linked to poisoning.

### Sample collection

- Biological Samples: Collection of biological samples such as blood, urine, and tissues (particularly liver and kidney) from the deceased or affected individuals, both ante-mortem and post-mortem specimens for comparison.
- Plant Material: Collection of samples of any suspected plants or plant-based products including roots, leaves, stems, seeds, or other parts that may contain toxins.
- Food and Beverage Samples: Collection of any food or beverage consumed that are suspected to be contaminated with plant toxins, ensuring that they are preserved in clean, sealed containers.

### Sample preservation

- Biological Samples: Freeze samples intended for chemical analysis to prevent decomposition/degradation and store them in appropriate containers to avoid cross-contamination.
- Plant Material: Dry plant samples for long-term preservation and store them in a cool, dry place away from direct sunlight.
- Food and Beverage Samples: Refrigerate perishable samples and freeze or dry samples intended for long-term storage.

### Chain of custody

Establishment and maintain a clear chain of custody for all collected samples. Document the handling, storage, and transfer of each sample to ensure its integrity and admissibility in court.

### Initial screening

Use rapid screening tests, such as ELISA, to quickly detect the presence of plant toxins in samples. While these tests provide initial indications, they may lack the specificity of more advanced analytical methods.

### Laboratory analysis: chromatography and mass spectrometry

Conduct a comprehensive analyses using GC–MS or LC–MS to identify and quantify specific plant toxins in biological and plant materials. Confirm the chemical structure of identified compounds using NMR spectroscopy, then compare the obtained results with control samples to ensure accuracy and reliability.

### Data interpretation

Interpret the analytical data in the context of the case, considering the potential sources of exposure and toxicokinetic of the involved plant toxins.

### Reporting

Prepare a detailed forensic report summarizing the findings, methods used, and conclusions. This forensic report should include information on the identification and quantification of plant toxins and their potential contribution to the observed effects.

### Expert testimony

If required, provide expert testimony in court to explain the analytical methods, results, and their forensic significance.

### Follow-up

Collaborate with medical examiners, toxicologists, and other experts to further investigate the implications of the plant toxin exposure. Also, monitor any potential public health concerns and implement preventive measures if necessary.

A comprehensive, well-documented forensic investigation including thorough sample collection, preservation, and analysis is essential for building a strong case in incidents involving suspected plant toxin poisoning. Collaboration among various experts and adherence to established protocols ensure the reliability of the findings in legal proceedings.

## Challenges in identifying plant toxins in forensic phytochemistry

Identifying plant toxins during postmortem examinations is a complex process presents several challenges due to the dynamic nature of postmortem changes, including degradation and metabolic alterations [26, 27]. Thus, it requires careful consideration of these factors for accurate toxicological assessments in cases involving suspected plant toxin poisoning. Forensic experts must navigate these challenges, select appropriate analytical methods, and collaborate across disciplines to provide accurate and reliable results. Here are some key challenges:

### Rapid postmortem changes

*Autolysis and Putrefaction* After death, enzymatic autolysis and microbial putrefaction can rapidly degrade tissues and substances present in the body [85], affecting the stability and detectability of plant toxins.

### Metabolism

*Postmortem Metabolism* Metabolic processes can continue for some time after death, potentially altering the chemical structure of plant toxins [86]. Metabolites formed postmortem might differ from those produced in the living organism.

### Biotransformation by microorganisms

Microorganisms present in the body can further metabolize plant toxins, complicating the identification process [87, 88].

### Sampling challenges

*Tissue Availability* The availability of intact tissues for analysis may be limited due to autolysis and putrefaction, making it challenging to obtain representative samples for toxicological analysis [89]. The distribution of plant toxins may also vary within tissues, requiring sampling from different body compartments for a comprehensive analysis.

### Toxin degradation

*Chemical Decomposition* Plant toxins may undergo chemical decomposition over time, especially when exposed to environmental factors such as temperature, humidity, and microbial activity [90]. This can lead to the loss of the parent compound or the formation of degradation products.

*Volatility* Some plant toxins are volatile and susceptible to evaporation, especially in cases where samples are not properly handled or stored.

### Postmortem interval (PMI) impact

*Time-Dependent Changes* The duration between death and the postmortem examination (postmortem interval) can significantly affect tissue integrity and the detectability of plant toxins. Earlier postmortem analysis generally yields more reliable results than delayed examinations [91].

### Forensic context

*Distinguishing Antemortem and Postmortem Exposure* Differentiating between antemortem exposure and postmortem contamination or generation of toxins can be challenging. Forensic toxicologists must carefully consider the circumstances surrounding the death and consider any potential sources of postmortem exposure [92].

## Geographic and environmental factors

The geographic distribution of certain plants and environmental conditions significantly influence the prevalence of plant toxins, adding a geographical context to forensic investigations of suspected plant poisoning [93]. Incorporating geographic and ecological considerations into forensic investigations is essential for understanding the factors that affect plant toxicity. By considering the unique regional environmental conditions and plant compositions, forensic experts can better interpret the circumstances surrounding suspected plant poisoning cases. This multidisciplinary approach enhances the accuracy and relevance of forensic analyses in diverse geographic settings.

### Endemic plants and toxins

*Regional Variability* Certain plants and their toxins are endemic to specific geographic regions. The prevalence of these plants may vary based on factors such as climate, soil type, and altitude [94]. Forensic investigators must be aware of local endemic plants and their toxins, as regional knowledge can aid in identifying potential sources of poisoning.

### Climate and soil conditions

*Temperature and Precipitation* Climate factors, including temperature and precipitation, influence the types of plants that can thrive in a given area. Certain toxins may be produced or accumulated more abundantly in specific climates [95]. Climate change may shift plant distributions, affecting both the prevalence and toxicity of certain species, which may require forensic investigations to adapt to these changing environmental changes.

*Soil Composition* Soil conditions, such as pH and nutrient levels, play a role in plant growth and toxin production [96]. Certain toxins may be more prevalent in plants growing in specific soil types.

### Altitude and elevation

*Altitudinal Gradients* Elevation affects plant distribution and, consequently, the prevalence of plant toxins [97]. Some plants may produce higher concentrations of toxins at different altitudes. Investigators should consider these variations in altitude when assessing potential plant poisoning cases.

### Biotic interactions

*Ecological Relationships* Biotic interactions among plants, such as competition and mutualism, can affect toxin production [98]. Competitive interactions may lead to increased toxin levels, while mutualistic relationships may result in toxin production as a defense mechanism. The introduction of invasive plant species can alter local ecosystems and impact toxin prevalence, potentially introducing new toxins or altering the concentrations of existing ones.

### Seasonal variation

*Plant Phenology* The timing of plant growth and reproduction stages, known as phenology, can influence toxin levels [99]. Certain toxins may be more concentrated in specific plant parts or seasons. Seasonal changes can affect the risk of toxin exposure, especially for individuals who rely on foraging or harvesting plants for food or medicinal purposes.

## Strategies for improving detection and analysis of plant toxins in forensic science

**Fig. 2:**
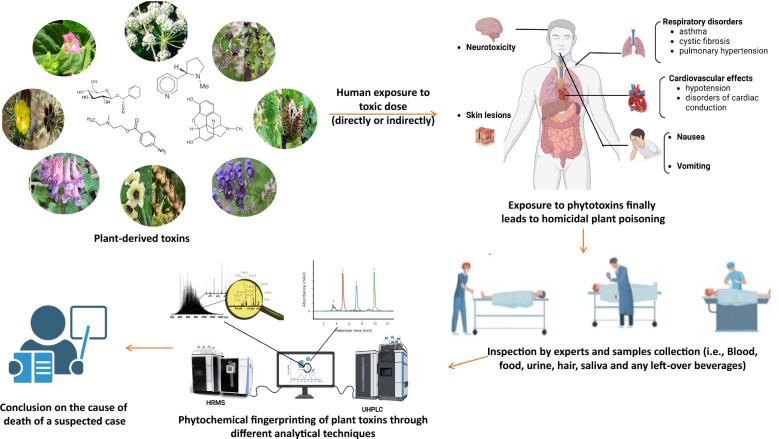
Forensic analysis workflow for plant toxicological impact assessment and toxin identification. Phytochemical fingerprinting of plant-derived Toxins of Forensic significance through Ultra-High-Performance Liquid Chromatography (UHPLC) and High-Resolution Mass Spectrometry (HRMS)

To enhance the detection and analysis of plant toxins, several advanced techniques and strategies can be employed to increase accuracy, resolution, and speed in forensic investigations (Fig. 2).

### High-resolution sophisticated chromatographic techniques

*Ultra-High-Performance Liquid Chromatography (UHPLC)* Utilizing UHPLC for improved separation and analysis of plant toxins [100]. This technology offers higher resolution, sensitivity, and faster analysis times compared to traditional chromatographic methods. It offers several advantages over traditional High-Performance Liquid Chromatography (HPLC), making it a preferred choice for many analytical applications. Here are some key advantages of UHPLC:

*Increased Resolution* UHPLC uses smaller particle sizes in the stationary phase, resulting in a higher efficiency and improved resolution, this allows for better separation of complex mixtures and the detection of closely eluting peaks [101].

*Higher Speed and Throughput* The use of smaller particles and increased surface area of interaction in UHPLC leads to faster analyte separation. This results in significantly reduced analysis times, leading to higher sample throughput. Shorter columns and higher flow rates contribute to quicker analyses [102].

*Enhanced Sensitivity* UHPLC systems typically operate at higher pressures, which allows for the use of smaller particle sizes without sacrificing efficiency. This increased pressure enhances sensitivity and allows for the detection of lower concentration analytes [103].

*Improved Peak Shapes* UHPLC produces narrower and sharper peaks compared to HPLC [104]. This is attributed to the smaller particle sizes, reduced band broadening, and increased efficiency, resulting in better peak shapes and improved accuracy in peak quantification.

*Reduced Solvent Consumption* The higher efficiency of UHPLC allows for the use of smaller elution volumes and reduced solvent consumption per analysis, making it both cost-effective and environmentally friendly [105].

*Compatibility with Mass Spectrometry (MS)* UHPLC is well-suited for coupling with mass spectrometry detection due to its higher efficiency and sensitivity. This combination enhances the capabilities for compound identification and quantification in complex samples. [106].

*Increased Productivity and Sample Throughput* The faster analysis times, improved resolution, and higher sensitivity contribute to increased overall productivity and sample throughput. Laboratories can analyze more samples in a given time frame, improving efficiency [107].

*Versatility in Column Lengths* UHPLC systems offer flexibility in choosing column lengths, allowing analysts to select columns tailored to the specific requirements of their separations [108]. This adaptability enhances the versatility of UHPLC for different applications.

*Improved Spectral Quality* UHPLC provides high spectral quality due to the narrow peaks it generates, which is beneficial for impurity profiling and compound identification [101].

*Compatibility with Existing HPLC Methods* UHPLC instruments are designed to be compatible with existing HPLC methods, making it easier for laboratories to transition to UHPLC without significant changes to their existing workflows [109].

### Mass spectrometry improvements

*High-Resolution Mass Spectrometry (HRMS)* Integration of HRMS for accurate identification and quantification of plant toxins [110], provides superior resolution and can distinguish between closely related compounds, reducing the likelihood of false positives. HRMS offers several advantages compared to conventional mass spectrometry techniques.

*High Mass Accuracy* HRMS provides high mass accuracy, allowing for precise determination of the mass-to-charge ratio (*m/z*) of ions, making it essential for identifying complex compounds [111].

*Increased Resolution* HRMS systems have higher resolving power, enabling the separation and identification of closely spaced peaks in a mass spectrum [112]. This enhanced resolution is valuable for analyzing complex mixtures and resolving isobaric compounds. The high resolution and mass accuracy of HRMS facilitate detailed structural elucidation of compounds. This is valuable in the characterization of unknown or novel compounds in research and discovery applications.

*Accurate Elemental Composition Determination* HRMS allows for accurate determination of the elemental composition of ions based on their accurate mass [113]. This is particularly useful in determining molecular formulas and reducing the likelihood of false identifications.

*Discrimination of Isotopes* HRMS can distinguish between isotopes with similar masses, providing additional information about the elemental composition of compounds [112]. This capability is especially valuable in isotopic labeling studies and metabolic profiling.

*Broad Analytical Range* HRMS instruments can cover a wide mass range, making them versatile for the analysis of various compounds, from small molecules to large biomolecules [114]. This broad analytical range enhances the applicability of HRMS in different fields.

*Flexibility in Acquisition Modes* HRMS systems offer various acquisition modes, such as full-scan, targeted, and data-dependent acquisition [115]. This flexibility allows researchers to adapt the analysis to different experimental needs, maximizing the information obtained from a single experiment.

*Reduced Interference* HRMS can reduce the likelihood of interference from co-eluting compounds due to its ability to resolve ions with similar masses [116]. This is particularly advantageous in complex samples where accurate identification of individual components is challenging.

*Compatibility with Chromatographic Techniques* HRMS can be coupled with various chromatographic techniques, such as liquid chromatography (LC) and gas chromatography (GC), allowing for the separation of complex mixtures before mass spectrometric analysis [117]. This enhances the specificity and sensitivity of the analysis.

*Advancements in Ionization Techniques* HRMS benefits from advancements in ionization techniques, such as electrospray ionization (ESI) and matrix-assisted laser desorption/ionization (MALDI), expanding its capabilities in different sample types and applications [110, 118].

Adopting these advancements in technology and methodologies can significantly improve the capabilities of forensic science in detecting and analyzing plant toxins. The integration of cutting-edge tools and collaborative approaches holds the potential to enhance the accuracy, speed, and reliability of forensic investigations involving plant-related poisonings.

## Conclusion and prospective directions

Plant-derived toxins as natural secondary metabolites, pose significant risks to human and animal health. The concept of phytochemical fingerprinting of plant toxins has been transformed forensic investigations by enabling precise identification and analysis of plant toxins. This innovative approach can be achieved through advanced chromatographic and spectroscopic techniques such as UHPLC and HRMS. These innovations will provide forensic scientists with even greater precision in identifying and characterizing plant compounds, strengthening the discriminatory power of botanical evidence. By analyzing the distinct profiles of plant toxins, forensic scientists can provide critical insights into crime scenes, identifying or eliminating the involvement of specific plants or toxins. As a result, forensic phytochemistry enhances the overall accuracy and reliability of criminal investigations, adding a new dimension to forensic science. The future of forensic phytochemistry holds promising avenues that can further revolutionize investigative processes and enhance the reliability of botanical evidence. Forensic phytochemistry is likely to extend its applications to environmental forensics. The analysis of plant evidence may contribute to investigations related to environmental crimes, such as illegal logging or pollution, providing a unique perspective on ecological impact assessments. Moreover, the future promises the development of portable and field-deployable analytical devices. These tools will enable real-time, on-site analyses by forensic investigators, accelerating the collection and interpretation of evidence, and supporting timely decision-making in the field. As forensic phytochemistry evolves, its role in both criminal and environmental investigations will undoubtedly expand, strengthening the pursuit of justice across a broader range of forensic contexts.

## Data Availability

All the data are available in the manuscript.
